# Supplementary material for: A community-based mentoring scheme for pregnant and parenting adolescents in Sierra Leone: Protocol for a hybrid pilot cluster randomised controlled trial
Source: PLoS One. 2024 Mar 25;19(3):e0294538. doi: 10.1371/journal.pone.0294538 (PMC10962822; doi:10.1371/journal.pone.0294538)
Supplement: S2 File — (DOCX) [file pone.0294538.s002.docx]

**STUDY PROTOCOL**

**2 Young Lives: mentoring teenagers for safer pregnancy and birth in Sierra Leone**

~~
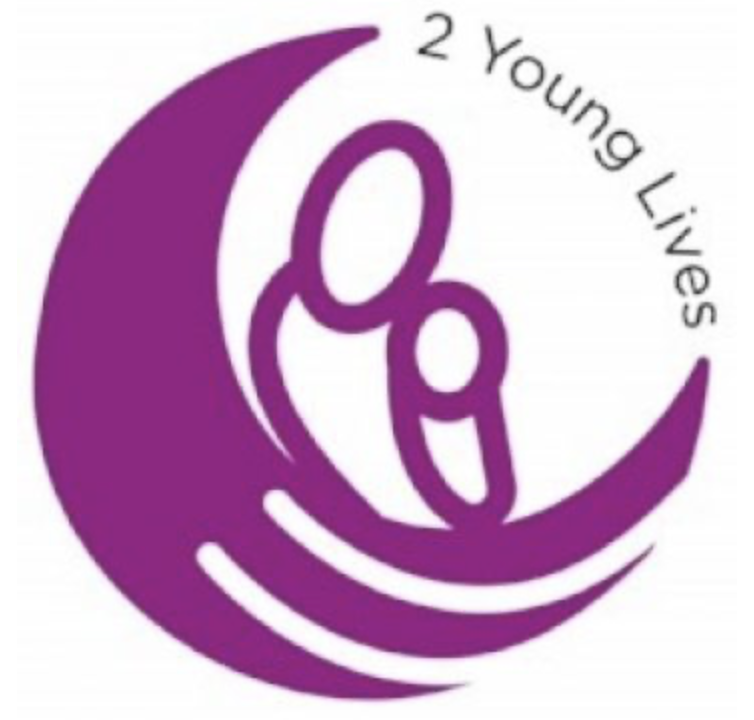
~~

~~
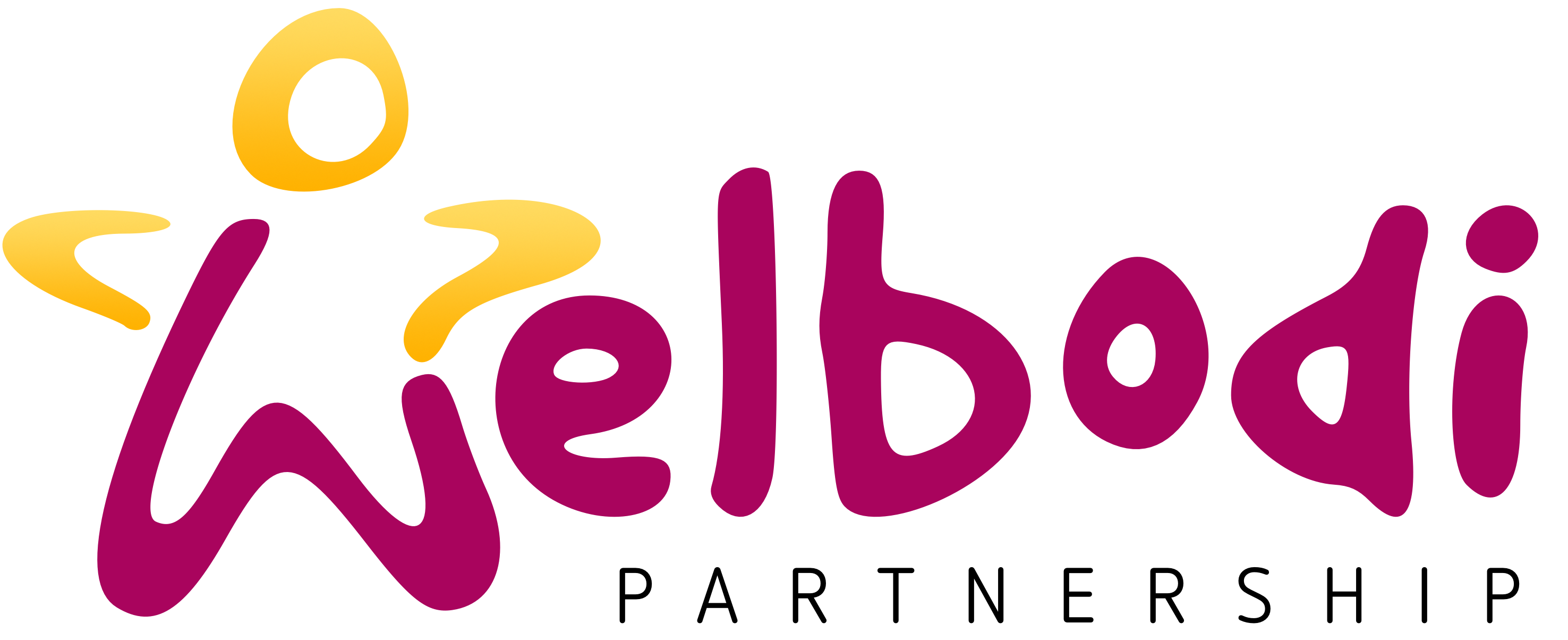
~~
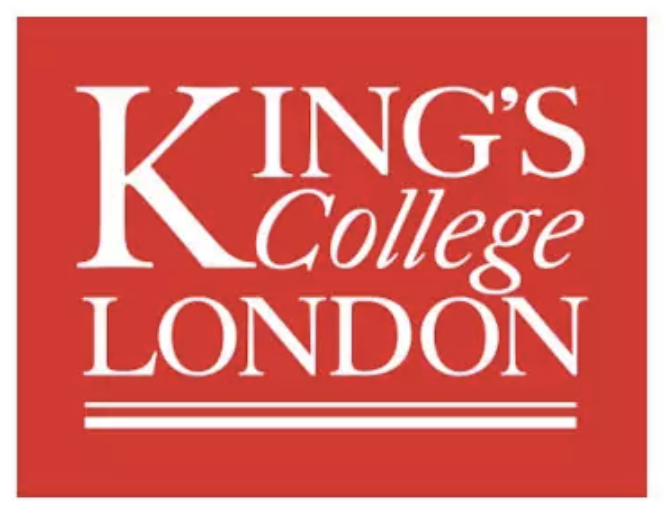

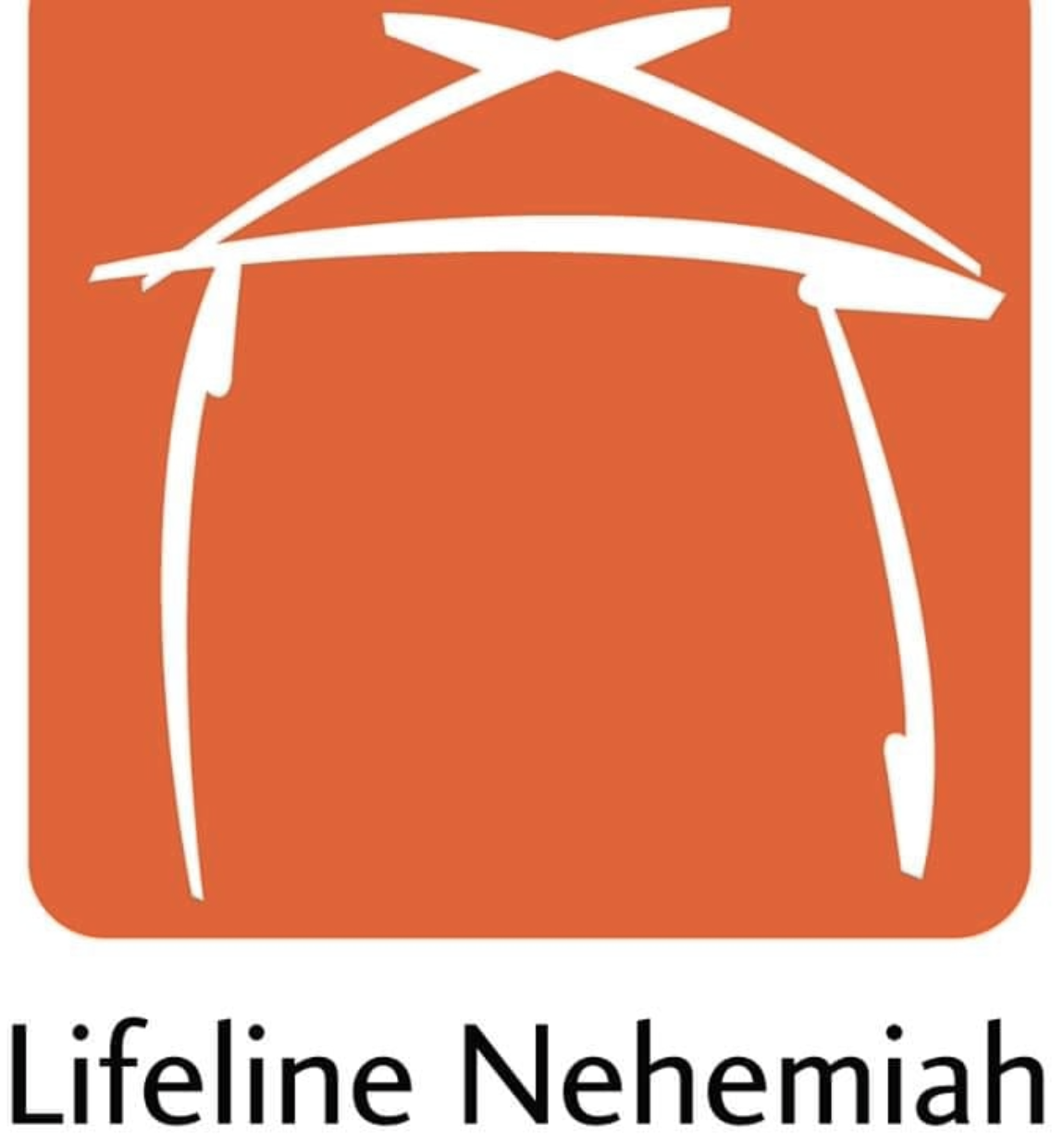

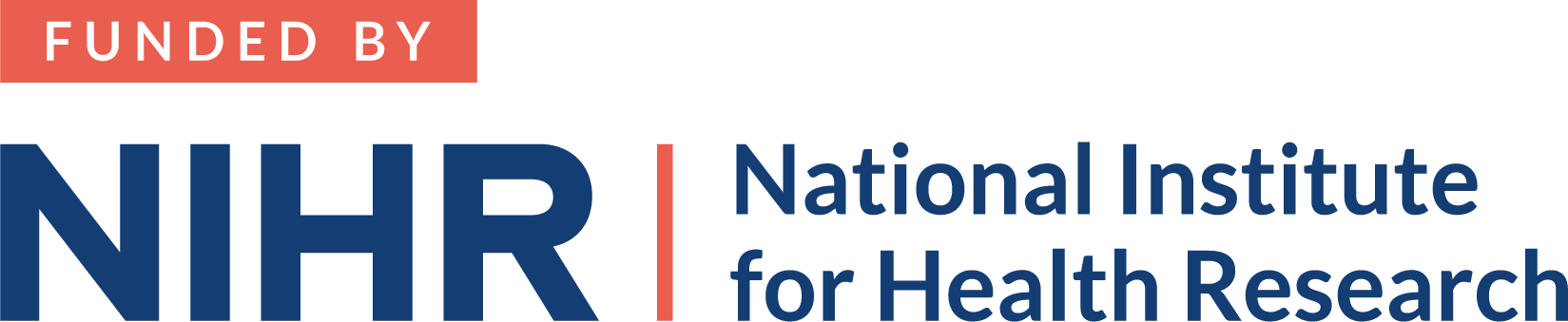


Table of Contents

[Research team 4](#_Toc88230731)

[Background and rationale for study 5](#_Toc88230732)

[How the 2YL intervention may work 6](#_Toc88230733)

[Study aim 10](#_Toc88230734)

[Study objectives 10](#_Toc88230735)

[Primary objective 10](#_Toc88230736)

[Secondary objectives 10](#_Toc88230737)

[Methods 10](#_Toc88230738)

[Study design, setting and population 10](#_Toc88230739)

[Inclusion criteria 12](#_Toc88230740)

[Exclusion criteria 12](#_Toc88230741)

[Control: usual maternity care 16](#_Toc88230742)

[Outcome measures 16](#_Toc88230743)

[Clusters description 18](#_Toc88230744)

[Data collection 18](#_Toc88230745)

[Primary and secondary pregnancy outcome data collection 18](#_Toc88230746)

[Process Evaluation: experiences and implementation 21](#_Toc88230747)

[Ethical considerations 21](#_Toc88230748)

[Mitigating risks to participants 23](#_Toc88230749)

[Data analysis 23](#_Toc88230750)

[Sample size calculation 23](#_Toc88230751)

[Proposed analysis 24](#_Toc88230752)

[Trial Management and oversight arrangements 25](#_Toc88230753)

[Pre-pilot trial 25](#_Toc88230754)

[Trial Registration 25](#_Toc88230755)

[Trial duration 25](#_Toc88230756)

[Data Management 25](#_Toc88230757)

[Research Governance 26](#_Toc88230758)

[References 28](#_Toc88230759)

[Appendix 1: 2YLs prospective sites 30](#_Toc88230760)

**Title**

| **Short Title** | 2 Young Lives (2YL) |
| --- | --- |
| **Aim** | To assess the feasibility and implementation of a mentoring programme for pregnant teenagers in Sierra Leone |
| **Sponsor** | Kings College London |
| **Funder** | NIHR Global Health Research Group |
| **Funding Reference Number** | NIHR133232 |
| **Duration (months)** | Funding period = 36 months, trial period = 24 months |
| **Grant activated** | 1 September 2021 |
| **Anticipated start date** | September 2021 |
| **Anticipated end date** | August 2024 |
| **Version Number and Date** | Version 2.1, 21 July 2023 |

# Research team

**NIHR Global Health Research Group Sierra Leone**

**Chief Investigators**

Prof Andrew Shennan, King’s College London, United Kingdom

Professor Sahr Gevao, University of Sierra Leone, Sierra Leone

**2 Young Lives Co-Principal Investigators**

Lucy November, King’s College London, United Kingdom

Dr Cristina Fernandez Turienzo, King’s College London, United Kingdom

**Co-investigators**

Prof Jane Sandall, King’s College London, United Kingdom

Mr Paul T Seed, King’s College London, United Kingdom

Dr Rachael Hunter, University College London

Dr Alexandra Ridout, King’s College London, United Kingdom

Dr Joan Shepherd, National School of Midwifery, Sierra Leone

Sister Margaret Mannah, Ministry of Health and Sanitation, Sierra Leone

Dr Francis Moses, Ministry of Health and Sanitation, Sierra Leone

Dr Andrew Leather, King’s College London, United Kingdom

Dr Sartie Kenneh, Ministry of Health and Sanitation, Sierra Leone

Mangenda Kamara, University of Sierra Leone, Sierra Leone

Prince Tommy Williams, Lifeline Nehemiah Projects, Sierra Leone

Dr Kate Bramham, King’s College London, United Kingdom

Dr Harriet Boulding, King’s College London, United Kingdom

Prof Lucy Chappell, King’s College London, United Kingdom

Dr Venetia Goodhart, Welbodi Partnership, Sierra Leone

Miss Kadiatu Bangura, Lifeline Nehemiah Projects

# Background and rationale for study

Maternal mortality in Sierra Leone is one of the highest globally (1,360 deaths per 100,000 live births in 2015)^1^ with teenage pregnancy a leading cause of death for mothers; teenage mothers are 40-60% more likely to die during childbirth.^2^ However due to ineffective maternal death reporting, this could be significantly higher. A household survey conducted in 2015 indicated a maternal death rate of 1 in 10 for under-18-year-olds in Eastern Freetown. A following study examined the causes of this high incidence of maternal death in teenagers and highlighted intersecting health and socio-economic vulnerabilities.^3^ Among key findings were that pregnant teenagers are often neglected by their families, particularly when being cared for by a non-parental adult, often sleeping on bare ground without a mosquito net, and are fed once a day in exchange for heavy domestic duties such as water collection and laundering, in many cases with little protection from ongoing gender-based violence. This lack of adult care or support often leads to delays in care-seeking or complete lack of antenatal and delivery care, putting teenagers at high risk of death from undetected pre-eclampsia, untreated infections, anaemia, lack of birth preparation, and common obstetric risks.^3^ To achieve the Sustainable Development Goals target of reducing the maternal mortality rate to less than 70 per 100 000 live births by 2030, adolescent girls are a priority group. ^4-6^

These findings led to the development of 2 Young Lives (2YL), a locally-designed intervention that provides mentoring from pregnancy through to one-year post-birth for women aged under-18. This encourages mentees to take up antenatal care and hospital birth, re-establish family connections where this is safe and appropriate, promotes health-seeking behaviour, provides practical advice about childbirth, parenting and contraception, and supports mentees to return to education or start vocational training. The 2YL scheme was piloted in 2017/2018 in an urban suburb of the capital, Freetown, and expanded to new sites in 2018, 2019 and 2021.^7^

An initial evaluation of the 2YL intervention compared outcomes of young women pre-intervention (n=86) with those of other women post 2YL intervention (n= 67).^7^ In the 2YL cohort of young women who had mentors there were fewer maternal deaths (0% vs 10%) and lower levels of perinatal mortality (6% vs 16%), and infant mortality (11% vs 26%). Nearly all women receiving the 2YL intervention gave birth with a skilled birth attendant (SBA) and breastfed for longer than 6 months; and more than two thirds were using contraception by the baby’s first birthday, with no second pregnancies. In terms of fidelity of the intervention, mentors are required to provide at least one opportunity per week for a confidential 1:1 meeting, however most provided almost daily contact. All of the 2YL young women successfully ran a small business with support from their mentor, allowing them to learn business skills, eat well throughout pregnancy and provide for their baby. They reported increased self-confidence, supportive peer relationships, and a high level of satisfaction with the mentoring scheme. Subsequent anecdotal evidence is also now showing that the community engagement strategy pursued as an integral part of the early implementation is influencing more widely on ingrained attitudes towards practices which perpetuate gender inequality such as school non-attendance in pregnancy and child marriage.

Based on this preliminary data, the 2YL mentoring scheme is promising; it has potential to reduce maternal, neonatal and infant deaths rates, and sustainably improve livelihoods. However, a pilot trial is needed to assess the feasibility of recruitment, retention, collection of data and evaluation design. Such a trial and process evaluation should draw on the six core elements considered in the updated MRC framework for evaluating complex interventions which include: interacions between intervention and context, theoring how the intervention may work, diversity of stakeholders perspectives in research, uncertainainties, refinement of the intervention and resource and outcome consequences of the intervention.^8^

# How the 2YL intervention may work

Overall, the aim of 2YL is that mentees and their babies survive birth and the postnatal period, and that these young women are empowered economically and socially to thrive. A theory of change diagram in Figure 1 in page 8 illustrates how and why the 2YL intervention may work in young women in Sierra Leone.

After community engagement with key community stakeholders, volunteer mentors are recruited and trained to support mentees across a number of domains. The 4-day manualised training programme uses discussion, role play and bespoke pictorial maternal health resources to learn and share important health messages. Key ingredients of the mentoring scheme include a weekly one to one meeting, promotion of health services uptake, monthly social gatherings for peer support and health discussions, advocacy and practical advice, support to start and run small businesses, and encouragement to return to education or start vocational training.

There are a number of mechanisms whereby being mentored could lead to improved health outcomes for mothers and their babies:

- Mentors ensure that girls access antenatal care, often paying for routine drugs when not available in the clinics, and directly observing the girls taking them. They ensure birth with a Skilled Birth Attendant, a proven life-saving intervention. In addition, the timeliness and quality of this care, and the quality of interaction with the girl in the health facility may be improved by the her being accompanied by a known and respected older woman in the community^9^; mentors are often allowed to be present for birth. Getting to know a health worker through monthly facilitated discussions and regular antenatal care could also increase girls’ confidence in the health service, increasing their likelihood of seeking care in labour or in an emergency. Similarly, having a trustworthy adult in their lives also allows mentees to ask advice of mentors and access care promptly.
- Girls are economically empowered through their small businesses, which could lead to a better diet in pregnancy, potentially addressing risk factors for maternal mortality such as malnutrition and anaemia. This economic empowerment could also allow girls to make independent decisions when to go to hospital as they could pay for their own transport^10^.
- After birth, mentors promote basic health and healthy parenting practices, such as uptake of immunisations, making healthy local weaning food, talking and singing to babies, recognition and first line treatment of common childhood illnesses and early health-seeking behaviour. This knowledge and support could promote behaviours which reduce risk factors for infant death.

Mechanisms by which mentoring might lead to young women being empowered economically, socially and educationally:

- Mentoring increases self-confidence, a sense of agency and the ability to make changes which allow mentees to make decisions and pursue personal goals. Having a trustworthy adult and peers going through a similar experience in their lives promotes better mental health, and encourages returning to education or training which leads to financial and social benefits to herself, this child and future children. In addition, educational empowerment about their human and health care rights could equip both mentors and mentees to report and escape gender-based violence, and advocate for themselves and each other at health facilities to receive more respectful and timely care. For girls whose babies die, the loving care given by the mentor can promote resilience and supports emotional wellbeing.
- Stakeholder engagement with teachers (October 2021) has led to an additional element to the intervention; the training of teachers to be school teenage pregnancy champions to action a new government policy aimed at reintegrating adolescent mothers back into school (the radical inclusion policy). The theory of change for this addition is that as schools become more aware of the rights and needs of pregnant girls and young mothers, this will make schools more enjoyable places to be, and girls will be more likely to stay in school with all its short- and long-term benefits for wellbeing.
- Educational bursaries: though not an intrinsic part of the intervention, 15 girls were given bursaries in September 2021 to return to school (with a small grant through KCL). During stakeholder engagement, it became clear how highly valued this was in terms of empowerment and wellbeing, with proposed mechanisms being their acceptance by family, and their sense of purpose and achievement. Additional partner funding will be sought to add this element to the trial intervention.

In addition, at a community level, respectful community engagement and involvement in the establishment of the scheme, including wider discussions about teenage pregnancy and child marriage can lead to wider socio-cultural changes to attitudes and practices. There is also some evidence of health workers being held to account when making demands for payment for maternity services.


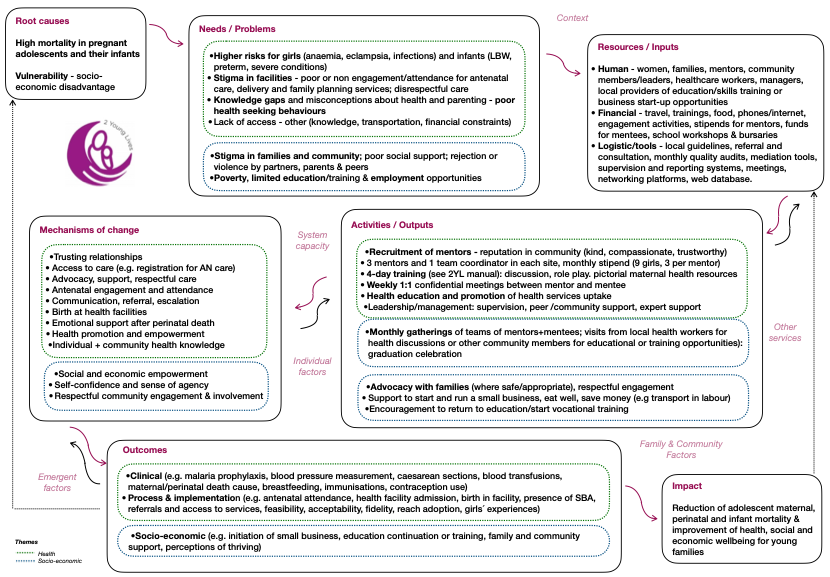


# Study aim

The overall aim of this pilot trial is to assess the feasibility and implementation of the 2YL mentorship scheme for young pregnant women in Sierra Leone to inform trial procedures for a subsequent fully powered cluster RCT evaluating the clinical, social and economic impact of the 2YL. This study will allow us to assess acceptability and feasibility of recruitment, retention, data collection, analysis and potential treatment effects, while also determining factors to optimize the intervention.

# Study objectives

Primary objective

To determine whether a pilot trial of the 2YL mentorship scheme for pregnant teenagers is feasible and to inform a future larger trial to evaluate the effectiveness of 2YL in improving pregnancy outcomes for mothers and babies in Sierra Leone.

Secondary objectives

To assess the integrity of study protocol including: recruitment, randomization procedure, data collection, retention and follow-up procedures, acceptability of intervention, primary outcome measure and sample size calculation, refinement of the intervention and training and supervision procedures, and estimation of resource use and costs.

To assess health, social, educational and economic outcomes of the 2YL intervention for young mothers and babies

To explore experiences, mentoring and thriving among young women receiving the 2YL intervention compared to those receiving usual care.

To evaluate the implementation, context and mechanisms of change of the 2YL intervention in order to understand the results and its potential impact.

# Methods

# Study design, setting and population

The design is a pilot cluster randomised controlled trial (cRCT) of the introduction of the 2YL mentoring adjunct to maternity care in Sierra Leone. The RCT will run in the communities served by the following 12 areas, representing a range of urban and rural settings:

| 1. Tombo town CHC | 7. Moriba Town (Lower Banta) CHP |
| --- | --- |
| 2. Mattru Town CHC | 8. Madina (Bum) CHC |
| 3. PAYCY's Clinic | 9. Senehun (Kamajei) CHC |
| 4. Taiama (Kori) CHC | 10. Moyamba Junction CHC |
| 5. Lengekoro CHP | 11. Heremakono MCHP |
| 6. Calaba Town CHC | 12. Yataia CHP |

The Standard Protocol Items Recommendations for Interventional Trials (SPIRIT) Figure adapted for the 2YL trial is shown below in Figure 1:

|  |  | **STUDY PERIOD** | | | | | | | | | | |  |  |
| --- | --- | --- | --- | --- | --- | --- | --- | --- | --- | --- | --- | --- | --- | --- |
|  | **Randomi-sation** | **Post-allocation** | | | | | | | | | | |  | **Close out** |
| **Timepoint*** | ***t_0_*** | ***t_1_*** | ***t_2_*** | ***t_3_*** | ***t_4_*** | ***t_5_*** | ***t_6_*** | ***t_7_*** | ***t_8_*** | ***t_9_*** | ***t_10_*** | ***t_11_*** | ***t_12_*** |  |
| Allocation | X |  |  |  |  |  |  |  |  |  |  |  |  |  |
| **Intervention** |  |  |  |  |  |  |  |  |  |  |  |  |  |  |
| *[2YLs+ usual care]* |  |  |  |  |  |  |  |  |  |  |  |  |  |  |
| *[Usual Care]* |  |  |  |  |  |  |  |  |  |  |  |  |  |  |
| **Assess-ments:** |  |  |  |  |  |  |  |  |  |  |  |  |  |  |
| *Composite primary outcome* |  | X | X | X | X | X | X | X | X | X | X | X | X |  |
| *Secondary outcomes* |  | X | X | X | X | X | X | X | X | X | X | X | X |  |

****_0_***, ***t_1-12_*** = Two months-time period

Figure 1. SPIRIT figure adapted for the 2YL trial

Sierra Leone is classified as a low-income country and has one of the highest mortality rates in the word, with a high burden of communicable and non-communicable diseases. It struggles to provide basic universal health coverage (UHC) and potable water and sanitation. Sexual violence and rape are common, and capacity to provide treatment to affected women and girls is extremely limited (State of Emergency over rape and sexual violence was declared in 2019). Sierra Leone also suffers an inadequacy of human resources for health; it has 2 skilled providers (doctors, nurses and midwives) per 10,000 population which is well below WHO’s health workforce targets for UHC and Sustainable Development Goal of 23.

Suboptimal maternal health care contributes to premature deaths, disability, and devastating spending in a country with an incipient financial crisis exacerbated by the 2014 Ebola epidemic (and further weakened during the ongoing COVID-19 epidemic). It has the highest maternal mortality rates in the world (1,360/100,000) with teens accounting for nearly 40% of all maternal deaths (more than two thirds caused by pre- eclampsia, haemorrhage and sepsis). Neonatal, infant and child mortality are also high (31.8, 83.5 and 11/1,000 respectively) and infants of mothers who die are up to 10 times more likely to die within their first 2 years. Antenatal attendance, access to skilled birth attendants and facility deliveries are low, and quality service delivery is often poor with disparities in access and availability to appropriate assessment and intervention, with delays in delivery, escalation of care and emergency maternity care.

Participants

Inclusion criteria

- Pregnant women aged under 18 at recruitment

Exclusion criteria

- There will be no exclusion criteria in terms of gestational age, as from an ethical and logistical standpoint, all young pregnant women should have access to the intervention, and pregnancy outcome data to be collected at an individual level.

Clusters and participant selection and enrolment

A flow chart for 2YL is presented in Figure 2. Twelve PHUs have been identified by local partners, and matched into pairs with corresponding delivery rates and distance to a referral hospital (Appendix 1). From these pairs, PHUs will be randomly allocated into intervention sites and control sites. With established approval from the Ministry of Health and Sanitation, health leaders at a district level will be approached by local research staff with an invitation for their PHUs to participate in the study. On the basis of local baseline data, we estimate that 36 women per PHU will participate, approximately 216 women per arm (Figure 1) (details of sample size calculations in page 23). In the event of changes to the PHUs serving a community during the study period (e.g. existing PHUs closing, new PHUs opening), we may invite a PHU to participate in the same randomised arm if it is serving the same community . Our rational is that this is a trial of a community-based intervention, we have allocated a treatment arm to the community, and the community are now accessing another facility. This approach will help us to maintain recruitment to both arms. For example, Yataya community has been randomised to the control arm. The new PHU that recently opened there will be automatically allocated to the control arm. We may also conduct a community based survey in those clusters with low numbers of pregnant adolescent girls to understand if numbers included in the trial are real or if there is overreporting of age in facilities.

In intervention sites, an established process of three community engagement visits will ensure buy-in of the intervention from key stakeholders and community members, and allow volunteer mentors to be recruited. Mentors are women selected based on being well known and respected in communities, having a reputation for kindness and being trustworthy, and having an existing interest in young women’s issues. This is followed by a 4-day manualised training course delivered by an experienced educationalist and in-country co-ordinator. Pregnant girls often self-refer or are brought by a friend or family member who has heard about the mentoring scheme from the community engagement activities. As per normal 2YL practice, the local team co-ordinator will meet with eligible pregnant girls and enrol them in the mentoring scheme as they come forward to a maximun of 36 per month (maximum number of girls that mentors can support per cluster due to limited resources). This help us to test the feasibility of the intervention in a pragmatic way while avoiding selection bias. An information leaflet will also be provided to girls in intervention clusters before enrolling in the mentoring scheme to help them understand the research, and what it involves to enable them to make an informed choice. Verbal consent will be obtained and recorded in the mentors’ registry book.

k=12

Randomisation

**Usual maternity care**

(k=6)

**2YL Mentoring scheme** + usual maternity care

(k=6)

**Routine data collection after birth**

(data collector)

**Routine data collection after birth**

(data collector)

**Routine data collection 6 weeks**

(PHU routine visit)

Invitation to participate in future

interviews, focus groups, photovoice

**Routine data collection 6 weeks**

(PHU routine visit)

Invitation to participate in future

interviews, focus groups, photovoice

**Recruitment, consent, data collection** ≈**3-6 and** ≈**9-12 months**

Interviews, focus groups, photovoice

**(Routine data collection at 1year** (routine PHU visit)

**(Routine data collection at 1year** (routine PHU visit)

Figure 2. Two Young Lives pilot study flowchart

Randomisation

The unit of randomisation is the trial area (or cluster), rather than individual woman. The sixteen trial areas will be allocated cluster numbers. Randomisation will be managed via a secure web-based randomisation and data management system hosted by MedSciNet with telephone back-up available at all times. Randomisation to intervention sites (2YL) and control sites (usual care) will occur in a 1:1 ratio. A minimisation algorithm will be used to ensure balance between the groups with respect to PHU use and distance to referral hospital. MedSciNet will write the randomisation program and hold the allocation code.

Intervention: 2YL mentoring scheme + usual maternity care

Women in the intervention group will receive the 2YL mentoring scheme provided as an adjunct to usual maternity care. Using the TIDieR reporting guidance^11^, a description of the intervention is as follows:

*Brief name*. 2 Young Lives mentoring scheme for pregnant teenagers and young mothers in Sierra Leone.

*Why? Brief rationale*. A qualitative study in Eastern Freetown in 2017 examined the causes of the high incidence of maternal death in teenagers. Among the key findings was that pregnant girls are often abandoned or neglected by their families, particularly when being cared for by a non-parental adult. It is common for pregnant girls to then stay with a more distant relative or with her boyfriend’s family, sleeping on bare ground without a mosquito net, and being fed once a day in exchange for heavy domestic duties such as water collection and laundering. This lack of adult care or support often leads to delays in care seeking or complete lack of antenatal and delivery care, putting girls at high risk of death from untreated infections and anaemia, lack of birth preparation, and common obstetric risks. It was hypothesised that having a mentor could mitigate against the risks of maternal and infant mortality and morbidity in pregnant adolescents and their babies. A review of the determinants of delivery service use identified low maternal age as a determinant for not accessing skilled care for delivery ^12^, and evidence from other community projects in Freetown indicate that having an advocate when accessing health care helps to reduce some of the barriers such as disrespectful care and informal charges. There is strong evidence that connectedness with an adult, be it a parent or a non-parental adult, appears to be foundational for adolescent health and well-being more generally ^13-15^, including delaying rapid second pregnancies ^15^. Stakeholder engagement in October 2021 identified lack of awareness in schools of the government’s new policy on encouraging pregnant teenagers to stay in school or return to education after birth, and lack of confidence to be able to cater for pregnant girls and young mothers. This has prompted the inclusion of a teachers’ workshop in the pilot trial.

*What? (Materials).* 2 Young Lives training manual details the content and delivery activities for a 4-day training course for mentors. 2 Young Lives teacher training manual for a 2-day workshop for teachers. Welbodi health cards are a set of bespoke pictorial representations of common scenarios and maternal / newborn health issues, laminated and collated into sets.

*What? (Procedures) and How? (Modes of delivery)*. Weekly meetings between mentor and mentee; going to a local health facility with mentee to register for antenatal care, and reminding to attend or attending with mentee for usual antenatal care; accompanying mentee to health facility when in labour or needing emergency care (or ensuring other birth partner is available according to mentee’s wishes); flexible support postnatally dependent on mentee’s support network; discussion with mentee to consider small business options and accompanying mentee to purchase first supply of goods; monthly site meetings with all mentors and mentees for social interaction, cooking, eating and group discussion using Welbodi health cards; visits from mentor to mentee’s family to advocate for family support, if safe and appropriate; a face-to-face training for teachers in each of the 8 intervention sites. Graduation celebration at the end of the mentoring scheme.

*Who provided?* Mentors and teachers are trained by an experienced educationalist with experience of training both cadres. Mentors are women selected from communities where mentoring will take place, recruited based on having a reputation for kindness and being trustworthy, and an existing interest in young women’s issues. Visitors at monthly meetings are HCPs from local health facilities, or other or other community members who talk about educational or training opportunities.

*Where?* Mentoring takes place in agreed venues in the community where the mentee feels comfortable and where mentee and mentor can have a confidential conversation. Monthly meetings take place in a location in the community where there is shaded space for a group of 15-20 to cook (outdoors on a fire) and eat; many villages have these shared spaces, or mentors’ compounds where there is tree-shade.

*When and how much?* Girls are recruited at any stage of pregnancy, and have at least weekly mentoring as described, until baby is one year old.

*Tailoring?* If mentees are identified who have physical or intellectual disabilities, the programme is tailored to their needs where possible. For example, by providing communication cards for a deaf girl to use in labour, or by monitoring a girl with a learning disability taking pregnancy medication.

*Modifications? 10. How well planned? 11. How well actual?* These elements will be reported on at points in the research cycle when formative evaluation of implementation is undertaken.

Usual or routine maternity care is described below and will be the same in both intervention and control groups.

# Control: usual maternity care

Usual maternity care in Sierra Leone is comprised of the following components:

- 8 antenatal checks
- Screening for Hb, VDRL, Sickle cell status, malaria, urinalysis, HIV, Hep B
- Sulfadoxine-pyrimethamine (SP) as an intermittent preventive treatment (IPT) against malaria
- Free insecticide-treated bednet (ITN) at first antenatal visit
- Single dose Albendazole (worm medicine)
- Nevirapine for prevention of mother to child transmission of HIV (for all)
- Ferrous Sulphate
- Tetanus Toxoid immunisations x 2.
- Birth with a skilled birth attendant in a health facility with referral by ambulance for emergencies
- Post birth observation for 24 hours (or more if needed)
- BCG and Oral Polio Vaccine 0 for baby before leaving hospital
- 3-7 days postnatal check for mother and baby
- Baby immunisations starting at 6 weeks postnatal. Defaulters followed up in community by community health workers
- Free contraception

# Outcome measures

**Primary feasibility outcomes**:

Eligibility, recruitment, retention and attrition rates

Randomisation, data collection and data completeness

Selection of most appropriate primary outcome measure and sample size calculation

**Primary clinical outcome**:

The primary clinical outcome is a composite of maternal death (all-cause, occurring during pregnancy, labour, or within 42 days of birth), stillbirth (born with no signs of live at or after 28 weeks of pregnancy, but before or during birth) and neonatal death (deaths among live births during the first 28 days). We will report the effect of the 2YL intervention on both the composite and its components for all woman/baby dies in the study; those who experience any one of the intervention components will be considered to have experienced the composite outcome. Evaluating the data collection strategy and tool is an important element of testing the feasibility of a larger trial; for example, whether following women up to one year is possible due to the mobility of this population.

**Secondary maternal outcome**

Secondary maternal outcomes will include, for example, Intermitent Preventive Therapy (IPT) for malaria, anaemia, Number of Tetanus Toxoid immunisations, caesarean sections, blood transfurions, birth at facility, sepsis, postpartum haemorrage, place of birth and attendant, uptake of contraception.

**Secondary perinatal outcome**s

Secondary perinatal outcomes will include, for example, gestational age, birthweight, Apgar, newborn resusciatation, breastfeeding within 1 hour, Kanguroo mother care,

6 weeks postnatal immunisation updake, infant death at 1 year.

**Other secondary outcomes**

Other secondary outcomes will include service use, processes and implementation outcomes including, for example, number of antenatal checks, number of antenatal checks with blood pressure measurement, number of referrals, postnatal check within a week of birth.

Fidelity: number of mentors and team coordinators recruited; number of training packages and workshops provided for mentors and teachers: number of weekly 1:1 meetings between mentors and mentee; number of monthly social gatherings and attendees; proportion of women being accompanied by mentees to register for antenatal care, during labour and to purchase first supply of goods; number of visits from mentor to mentee’s family.

Acceptability: satisfaction with differet components of the intervention among young girls, mentors, healthcare providers and local stakrholders

Reach: number, proportion of participating young women when compared to the site population included

Adoption: engagement and updake of the mentoring scheme: proportion of site areas providing the mentoring scheme; number of sites interested in implementing the mentoring program.

Women’s experiences of mentoring and care (e.g., respectful care, access, trust, system responsiveness, quality and safety, attitude to and uptake of contraception in the postnatal year) *and* economic and social progress/wellbeing (e.g measures, return to education, set up a business, perspectives of thriving)

Mechanisms of change for health outcomes (e.g. access to care, advocacy, support, respectful care, engagement, referral and escalation, health promotion and empowerement, relationships) and for social and economical wellbeing (e.g. social and economic empowerment, self-confidence, sense of agency, trustworthy adult and peers, respectful community engagement with families)

# Clusters description

Each cluster will be described according to the number of primary, secondary and tertiary facilities and their referral distances. Details on staffing levels and availability of key resources (e.g. neonatal care, blood transfusion, magnesium sulphate) will be collated. In addition, major changes to the trial catchment, such as changes to infrastructure, policy, patient payment requirements or environmental conditions (e.g. physical and cultural context around teenage pregnancy, management of complications, escalation of care, referrals systems) will be reviewed systematically.

# Data collection

Primary and secondary pregnancy outcome data collection

Outcome data will be recorded over the study period and will be collected at an individual level. Recent stakeholder engagement has identified the routine data collection tools used to collect routine data (ANC attendance register, birth register, referral register, 6-week postnatal check appointment book). At some PHUs, an additional ‘adolescent book’ is in use to record ANC registration of under-18-year-olds and associated birth outcomes). Regular visits to PHUs by data collectors will use these to identify under-18-year- presenting at the health facility (e.g. registering for ANC, delivering their babies in the PHU or in a referral hospital, or attending the PHU for the 6-week postnatal appointment) and will allocate each a unique study number which will be recorded in a book that is kept securely in the clinic in the same way as the clinic health registers. Routine practice is for women who do not return for infant immunisations at 6 weeks post-natal to be followed up in the community by a community health worker (CHW). This will allow for maternal deaths in the 42 days after birth, and for neonatal deaths up to 28 days to be included as part of the routine data collection. At the 6 weeks routine postnatal appointment, young mothers in both arms will also be given the option to individually enrol and participate in the sub-studies (e.g. qualitative interviews, photovoice), in order for qualitative data to be collected at around one year (e.g. experiences of care, acceptability, use of contraception, plans to return to education or training, business set up). As mentioned in the application, for those agreeing to participate in the sub-studies, explicit consent will be gained.). Adolescent girls identified by the research assistant who have a disability and/or recently completed the mentoring programme at pre-trial sites may also be invited to take part in the photovoice project. One year infant routine data will be collected from the infant immunisations registry.

The following table represents various scenarios and strategies for data collection in these scenarios:

| Scenario | Strategies to mitigate lost data |
| --- | --- |
| Girl attends ANC, gives birth at PHU and attends 6-week check at PHU | N/A |
| Girl attends ANC but does not give birth in the PHU (maternal death, stillbirth or neonatal death may be missed) | Most likely girl has moved to another area    Liaise with referral hospital to cross-check birth register. If not there;  Data collector to visit address in AN register. If not found;  Connection with midwife investigators from MDSR teams in intervention sites to detect any maternal deaths in community. |
| Girl does not attend ANC but comes to PHU in labour | N/A. |
| Girl attends ANC at PHU but is referred for antenatal complication (very young, very small, sickle cell, twins, breech etc) (maternal death, stillbirth or neonatal death in referral hospital may be missed) | Since she will not necessarily arrive by ambulance, she will be harder to track than emergency transfer in labour.  However, we will liaise with da or HCW at referral hospital to cross-check birth register. |
| Girl attends ANC at PHU but is transferred in labour as an emergency (maternal death, stillbirth or neonatal death in referral hospital may be missed) | Through regular liaison with the referral co-ordinator in nearest referral hospital will find outcome data on girls who are referred. |
| Girl gives birth at PHU but does not attend for 6-week PN appointment (neonatal death, postpartum maternal death might be missed) | CHWs are routinely sent out to check on women who default at 6 weeks. |

Data collection at intervention and control sites will be done by data collectors. Data collectors will be responsible for entering the data anonymised onto MedSciNet, the study-specific management system. To prevent double counting, the research assistant will use their regular visits to ensure no patient is counted twice according to patient identifiers (e.g., in the community and at facility or for maternal and neonatal death).

We are confident, (through interaction with local community workforce and current local initiatives to ensure maternal death reviews), that it is unlikely that most primary outcome events will be missed. We will apply a flexible approach to individualise our methodology according to local capacity and CHW workforce infrastructure.

Consistency and quality of source data will be monitored by the research assistant and CIs. MedSciNet allows for extensive monitoring and query processing features, as well as a comprehensive alerting system to identify missing data. Fields including ‘limits’ will be used to avoid entry of erroneous data. The research assistant will monitor data entry by each data collector continuously on MedSciNet. They will be able to filter and identify any problem areas and validate 10% of the source data to check for transcription errors. It will be possible to review the audit trail at individual levels.

Process Evaluation: experiences and implementation

In line with the current best practice in implementation research, the team will undertake a process evaluation to understand variations in the impact of the intervention on outcomes of interest and to contextualise findings. This will be guided by the Medical Research Council (MRC) guidance for the evaluation of complex interventions and will use mixed methods to assess implementation outcomes and identification of mechanisms and contextual factors that may influence variation in outcomes and experiences across multiple stakeholder groups, and at different stages of implementation. This offers a 360-degree evaluation, which considers needs and perspectives that typically differ between stakeholder groups and can vary over time.

Both quantitative and qualitative data on processes and implementation will be collected from monthly checklists, separate focus groups with a purposive sample of mentors, PHU healthcare staff and other community stakeholders such as teachers and community elders (n≈4), and semi-structured interviews with young women in control and intervention sites (n≈40). If appropiate, a family member or a friend will also be invited to participate in an interview. Women will be offered the opportunity to be one-to-one (interviewer and participant) or one-to-two (interviewer and participant with chosen family members/friend). Thus, if appropriate, family member/friend can be invited to the interviews as social support is culturally patterned and could play an important part in the mechanisms of the intervention, particularly in women’s engagement with care. Interview administered wellbeing measures will include, for example, PHQ-9, the flourishing scale for adolescents, women’s empowerment scale. A small sub-group of participants (n≈6-10) will also be invited to take part in the photovoice pilot project^18^.

Considering how different contexts may affect implementation, as well as the intervention itself, will allow a flexible approach to future scaling up based on this understanding. The following table indicates possible research questions which will be refined in forthcoming stakeholder workshops and will be discussed in focus groups discussions and interviews:

| Context item | Possible interaction with implementation | Specific examples |
| --- | --- | --- |
| Geographical | Rural vs urban settings?  Seasonality – how do the seasons impact on the implementation of 2YL. | In rural settings, how are mentees supported with small business option when there is no local market?  Do monthly gatherings still take place in the rainy season?  Can mentoring mitigate the 2^nd^ delay when roads are impassable in the rainy season? |
| Socio-cultural | Family make-up: living with birth parents / relatives; married (child marriage) vs unmarried  Tribal, religious, ethnic norms and beliefs | Where child marriage is common, how does being married impact on the empowerment 2YL confers?  Is there a reluctance from husbands for married girls to enrol in 2YL? How can this be overcome?  Are there characteristics of tribal, ethnic and religious groups that impact on the acceptability of 2YL? (Various elements such as business, education, contraception) |
| Socio-economic | Economic status of family, especially if girl’s mother is single, or she is living with relatives.  Economic status of area. Some villages have higher levels of poverty.  Seasonal poverty, especially with climate change effects on rainfall. | What are the factors which make some girls’ businesses succeed and others fail? Can 2YL be adapted to mitigate this?  Do girls from poorer backgrounds benefit more from the small business?  Are there any risks associated with being given money for the small business?  How do seasonal effects impact? |
| Ethical | The level of stigma in community. Does this reduce over time?  Disrespectful care | How does 2YL affect the stigma associated with teenage pregnancy? Anecdotal examples of chief publicly speaking out against child marriage, and promoting return to education.  What are the key elements of the community engagement which lead to stakeholder buy-in?  How does 2YL impact at meso level as well as micro level? How do ‘new’ sites and ‘old’ sites differ in the impact 2YL has on  Does 2YL mitigate disrespectful care? |
| Political / legal | Government policies around teenage pregnancy and child marriage; high levels of law enforcement; new strategy | How do government policies ‘hands off our girls’ and ‘radical inclusion’ (in education) interact with 2YL and the willingness of communities to engage?  Is the law counter-productive in that it keeps girls away from ‘authority figures’ (health staff), for fear of being targeted / blamed / having their partner or parents arrested? |

# Ethical considerations

Investigators will ensure that this study is conducted in accordance with the current principles of the Declaration of Helsinki (October 2008). The conduct of this study will be in full compliance with the relevant regulations and principles of Good Clinical Practice. The study will only start after gaining Institutional level consent and ethical approval from the local committee in Sierra Leone.

- **Primary and secondary clinical outcome data:** Primary outcome data will be collected individually, and we believe facility-based consent is appropriate considering the following, and will be requested from the ethics committee:

- The 2YL intervention is not a clinical intervention, is of minimal risk, and does not adversely affect the rights and welfare of the individual women.

- The intervention is delivered at the level of the trial area (cluster), rather than the level of the individual woman (the unit of randomisation is the trial area, not the individual woman)

- The requirement to seek consent from all young girls within each trial catchment area may make the trial unfeasible, particularly considering that individual woman will be unidentifiable by the research assistant at centralised data collection.

- **Process evaluation (including women’s experiences) data:** Informed written consent or thumb print will be sought by the research assistant for qualitative interviews and focus group discussions with healthcare workers, mentors and young women and their families. A pre-tested information sheet will be provided and discussed with participants and reasonable time (24 hours) will be given to consider this information before being asked to consent to participate. Potential participants will be asked whether they would like the information sheet to be read out or translated verbally into the local language (the recognised written language is English, and the information sheets will be in English; however, for verbal explanations, local languages will be used throughout).

**-** For the health workers focus groups, the research assistant will visit the PHUs, speak to the midwife in charge, and leave information sheets allowing a period of one week for potential participants to consider participation.

**-** For young women and their families where there is no assumption of literacy, a verbal explanation of the study will be provided in Krio or other local language, and give out information sheets which will be pre-tested. Consent will be sought at the start of all interviews and focus groups, with the information sheet being explained again, with opportunity for questions. For participants under 16, written consent will be sought from their parent or guardian where possible, using a personal approach by the research assistant to explain the research and the information sheet. Assent will be sought from the under 16-year-olds.

All participants being asked to provide personal sensitive data will be asked to give explicit consent for the collection and use of such data using the standard wording of the Data Protection Act statement. Participants will be fully informed on the Information Sheet of the intention to re-use or retain data for purposes other than the trial report, e.g. journal articles. Participants will be given the opportunity to withdraw from participation at any point both during the data collection and for up to four weeks following data collection.

Where eligible participants are present prior to a focus group starting (e.g., by a girl bringing an eligible friend along), consent will take place just prior to the focus group on the grounds that bringing the participant back on another occasion may disadvantage the participant economically or educationally.

Mitigating risks to participants

Talking about past abuses, bereavements or near-death experiences puts participants at risk of psychological stress or anxiety. To mitigate this risk, it will be clearly explained at the beginning of the focus group or interview that should the participant wish to stop the interview or step out of the focus group, that this is acceptable and that the research assistant can accompany them if they wish. If a participant becomes visibly upset, they will be encouraged to take a break from the interview or focus group. In both cases, their willingness to participate after a break should be reassessed. At the start of the interview or focus group, participants will be told that if they feel they would like some more support or an opportunity to talk in a safe place after the interview or focus group, they will be given a phone number to call to get help to arrange this. Young people may disclose illegal sexual activity - for example with a teacher or other adult in a pastoral role to the young person. In these situations, LNP safeguarding guidelines will be followed. In the case of disclosure of these and other harmful behaviours, details of various organisations offering specialist support will be offered to the participant.

Names and geographical locations will be removed from the transcription in order not to identify participants based on family or social group or address. If identification is still possible from other descriptive factors, these will be removed.

# Data analysis

# Sample size calculation

Sample size estimation has been provided by the trial statistician, Mr Paul Seed, co-investigator and Reader in Medical Statistics, KCL. Based on experience with some of the proposed trial areas and global maternal health literature relevant to these settings^3,6^, we estimate the risk to adolescent pregnant women in Sierra Leone of experiencing a primary outcome (one of maternal death or stillbirth or neonatal death with no double counting) to be approximately 20% of all adolescent women. We also estimate a modest intra-cluster correlation of 0.02 (sites similar but not identical, and a simple exchangeable autocorrelation structure based on previous experience in Sierra Leone)^6^.

Prior to full proposal development, the 2YL team visited a typical peri-urban PHU similar in size and distance to referral hospital to trial sites. At least 16% of all deliveries among teens resulted in maternal death, stillbirth or early neonatal death. It was not possible to include late neonatal deaths up to 28 days. In addition, girls who were referred to a referral hospital in an emergency were not included in these numbers. We believe that this data can match the indicative estimate of 20% of girls experiencing a primary outcome.

This study is planned as a pilot, with main stated aim to demonstrate the feasibility of a fully powered study, and to help with its design. But for the purpose of the power calculation (intention to treat and not per protocol), an assumption of at least 42 deliveries per site has been made, with 20% having primary outcome composite events during the trial duration.  These numbers will provide 84% power to detect a 52% relative risk reduction (RRR) of the primary outcome (from 20% to 9%) assuming a models ICC 0.02.

# Proposed analysis

We will undertake two statistical analyses: one pragmatic, intention to treat analysis (ITT) to compare intervention and control clusters that include all adolescent girls as originally allocated after randomization; and a complementary per protocol (PP) analysis to compare intervention and control clusters that includes only those girls who completed the intervention originally allocated. The CONSORT guidelines for reporting of parallel group RCTs recommend that both ITT and PP analyses should be reported for all planned outcomes to allow readers to interpret the effect of an intervention. It is not possible to blind clusters to interventions they receive, but outcome assessments will be blinded.

The main analysis will be pragmatic and by logistic regression adjusting the standard errors for clustering. This gives equal weight to each woman. A secondary analysis using random effects generalised least squares (GLS-RE) will also be attempted, although past experience is that the model might not converge to give any useable results. We will adjust for important baseline differences that might be related to outcomes (e.g. parity, gestation at first antenatal visits). As individual-specific data will be used there is a potential for missing data; but the main impact of missing data would be to invalidate the randomised comparison if there was unequal dropout between the two arms, leading to potential boas. Multiple regression, as described, would correct for any such bias.

Qualitative data from interviews with women and focus groups discussions with mentors, healthcare providers and stakeholders with be analysed using Braun and Clarke's six-phase approach to thematic analysis^18^. In brief, this includes familiarisation with the data, generation of initial codes, the searching for and review of themes, naming and offering explanations for each theme, and lastly producing a report. This practical analytic approach involves inductive coding practices which are both consultative and initially open^19^, and thus helpful to explore the perspectives of different research participants, highlighting similarities and differences, and generating unanticipated insights^18^.

We will pilot the use of photovoice as a participatory research methodology using small group discussions of photographs with young women. Photovoice is a photographic technique developed by Wang and Burrithat^20^ that puts cameras into the community members’ hands to help them to record, reflect upon, critically dialogue and share knowledge about their perspectives and priorities to reach policy makers^20,21^. These are all central elements of empowerment, a concept that is intertwined with the photovoice approach from its original conceptual underpinnings to its implementation^22^. While photovoice aims to foster social change, it can also enhance community engagement, increase awareness of community resources, and improve collaboration with research partners to help reduce health inequalities^23,20^ Photovoice has previously been used in vulnerable communites in Sierra Leone^24^ and other settings^-25-28^ Thus, this approach offers a unique opportunity and gain understanding about the social and cultural factors that may facilitate potential scale up and sustainability of the 2YL mentoring scheme in young women, that other research methods such as surveys and interviews may not fully capture.

Feasibility of health economics

It would not be appopiate to conduct a full economic evaluation for a pilot study designed to estimate the parameters needed to design a future definitive trial - should not provide estimates of treatment effectiveness because the samples often have no or limited power to reliably detect between group differences. Thus, the focus of health economics in this pilot trial will be limited to developing or refining service use schedules and other measures of outcomes (i.e. piloting of a metric for risk-benefit assessment previously used in LMICs).

We will explore the resource use associated with the mentoring scheme and savings or other service use impacts (e.g. examining clinical records, based on clinical opinions) and will also estimate costs associated with the delivery of the scheme (components will include, e.g. recruitment and training of mentors, stipends, bursaries for girls, social gatherings). Cost consequences analyses will be used to present the results from this pilot study, as the costs and effects observed can also be used in value of information modelling to determine whether the cost of a definitive RCT is worthwhile.

# Trial Management and oversight arrangements

Pre-pilot trial

From September 2021 (start of grant) to January 2022, we will employ a research assistant and a PhD student, conduct community engagement visits, create contacts at each trial site, secure ethical approval at each trial area institution, and confirm the data collection procedures.

Trial Registration

The trial will be registered with https://www.isrctn.com following ethical approval.

Trial duration

Randomisation will start in March 2022, community engagement, recruitment and training of mentors during April and May 2022 and intervention implementation will start in May/June 2022. Latest data collection will be June 2024.

Data Management

Source data will be kept in paper-based databases kept in secure areas of each facility and of the implementation partners Welbodi and Lifeline Nehemiah Project. Only healthcare providers, research assistants and trial coordinator will have access to these databases. All data entered on the MedSciNet database will be automatically stored and backed-up.

King’s College London holds responsibility for ensuring participants’ information is kept confidential. All paper documents will be stored securely and kept in confidence in compliance with the Data Protection Act (1998). Outcome data will be entered onto the electronic forms and undergo a number of validation checks to verify the validity and completeness of the data captured; the data will be automatically transferred for storage in the electronic anonymised database held by MedSciNet on behalf of the Sponsors. Lucilla Poston, KCL, is registered for use of personal data with Information Commissioner’s Office. The hosting company, MedSciNet has a ISO 27001 certificate. MedSciNet's solutions conform to relevant FDA, NIH, and HL7 standards, guidelines, and recommendations. The MedSciNet database will be maintained ‘live’ for at least one year following the end of the trial. A copy of the database is then kept on the KCL Server for an indefinite period and is the responsibility of the unit statistician, regulated by the Joint Clinical Trials Office. Backups of the MedSciNet central database are put into fire safe vaults for a minimum 10 years storage. If needed data can be transferred to DVD disks for even longer storage.

The research assistant will monitor security and confidentiality on a monthly basis. If a breach of confidentiality or security is identified, this will be fed back to the CIs and/or co-investigators who will resolve the issue quickly. Once the data has been transcribed to MedSciNet, risks to confidentiality and security will be minimal, owing to the inbuilt security provisions. All data from the MedSciNet is stored on highcapacity servers that are operated by an external company. Servers are stored in locked rooms, with extra measures for security including auxiliary power through UPS and diesel generator; system monitoring 24x7; physical surveillance with video and infra-red cameras. A tape backup system is used for backing up the database. The tape backup system with tape loaders, central application servers and repository database servers are all physically away from the centre where the servers are stored.

When data are sent for merging with master databases, the research staff will also keep a copy of the file sent (in Stata form) on their own KCL drive. Data are stored on secure database and file servers on a high- speed network. Servers are stored in a secure locked server room with restricted access to key Centre staff. Server room is protected by alarms, motion detection cameras and secured via 2 separate keypads. The server room is monitored for environmental changes, availability and for any unapproved access.

Research Governance

**Sponsor**: King's College London

**Project Management Group (PMG)** - the trial will be run on a day-to-day basis by this group, which reports to the Trial Steering Committee (TSC), which in turn is responsible to the sponsor. Members include Cristina Fernandez turienzo, Lucy November, Mangenda Kamara, Jane Sandall, Andrew Shennan, Alexandra Ridout. The rest of co-investigators will be informed of all decisions during these meetings and can join virtually as they wish. The core PMG will convene every week, with all co-investigators convening every months to review progress, troubleshoot and plan strategically.

**Independent Trial Steering Committee (ITSC)** – An ITSC will include will be part of the NIHR Global Health Research Group and will will include a chair, at least three other independent members, a CEI representative(s), and the Chief investigators. The TSC will review the progress of the trial.

# References

1. WHO, Trends in Maternal Mortality: 1990 to 2015. Estimates by WHO, UNICEF, UNFPA, World Bank Group and the United Nations Population Division. Executive Summary.
2. UNFPA, National strategy for the reduction of adolescent pregnancy and child marriage, 2018-2022.
3. November L, Sandall J. ‘Just because she’s young, it doesn’t mean she has to die’: exploring the contributing factors to high maternal mortality in adolescents in Eastern Freetown; a qualitative study. Reproductive Health, 2018. 15(1): p. 31
4. Vogel JP, Pileggi-Castro C, Chandra-Mouli V, et al. Millennium development goal 5 and adolescents: looking back, moving forward. Arch Dis Child 2015;100 Suppl 1:S43–7
5. Nove A, Matthews Z, Neal S, et al. Maternal mortality in adolescents compared with women of other ages: evidence from 144 countries. Lancet Glob Health 2014;2:e155–64.
6. Li, Zhihui, et al. "Maternal healthcare coverage for first pregnancies in adolescent girls: a systematic comparison with adult mothers in household surveys across 105 countries, 2000–2019." BMJ Global Health 5.10 (2020): e002373.
7. Kamara M, November L. 2 Young Lives: mentoring teenagers for safer pregnancy and birth; Project report 2020. 2 Young Lives website. June 2020. Accessed October 05, 2021. <https://2younglives.org/wp-content/uploads/2020/07/2YL-2020-report-final.pdf>
8. Skivington K, Matthews L, Simpson SA, Craig P, Baird J, Blazeby JM et al. (2021) A new framework for developing and evaluating complex interventions: update of Medical Research Council guidance. *BMJ*;374:n2061
9. Lavender T, Bedwell C, Kasengele CT, et al. Respectful care an added extra: a grounded theory study exploring intrapartum experiences in Zambia and Tanzania. BMJ Global Health 2021;6:e004725. doi:10.1136/ bmjgh-2020-004725
10. Actis Danna V, Bedwell C, Wakasiaka S, Lavender T. Utility of the three-delays model and its potential for supporting a solution-based approach to accessing intrapartum care in low-and middle-income countries. A qualitative evidence synthesis. Global health action. 2020 Dec 31;13(1):1819052.
11. Hoffmann T C, Glasziou P P, Boutron I, Milne R, Perera R, Moher D et al. Better reporting of interventions: template for intervention description and replication (TIDieR) checklist and guide BMJ 2014; 348 :g1687 doi:10.1136/bmj.g1687
12. 4.Gabrysch, S. and O.M. Campbell, Still too far to walk: literature review of the determinants of delivery service use. BMC pregnancy and childbirth, 2009. 9(1): p. 1.
13. Coinco, E., A glimpse into the world of teenage pregnancy in Sierra Leone. UNICEF, Freetown, Sierra Leone, 2010.
14. Schaffer, M.A. and N. Mbibi, Public Health Nurse Mentorship of Pregnant and Parenting Adolescents. Public Health Nursing, 2014. 31(5): p. 428-437. 7.Sieving, R.E., et al., Youth–Adult Connectedness:: A Key Protective Factor for Adolescent Health. American Journal of Preventive Medicine, 2017. 52(3, Supplement 3): p. S275-S278.
15. Onuoha, F.N. and T. Munakata, Inverse association of natural mentoring relationship with distress mental health in children orphaned by AIDS. BMC psychiatry, 2010. 10(6): p. 1-8.
16. Black, M.M., et al., Delaying Second Births Among Adolescent Mothers: A Randomized, Controlled Trial of a Home-Based Mentoring Program. Pediatrics, 2006. 118(4): p. e1087-e1099. 10.Shamed and blamed.
17. Vousden N, Lawley E, Nathan HL, Seed PT, Gidiri MF, Goudar S et al. Effect of a novel vital sign device on maternal mortality and morbidity in low-resource settings: a pragmatic, stepped-wedge, cluster-randomised controlled trial. The Lancet Global Health. 2019 Mar 1;7
18. Clarke V, Braun V. Teaching thematic analysis: Overcoming challenges and developing strategies for effective learning. The psychologist. 2013 Jan 1;26(2).
19. Braun V., Clarke V., Hayfield N., Terry G. Handbook of Research Methods in Health Social Sciences. 2019. Thematic analysis; pp. 843–860
20. Wang C, Burris MA. Photovoice: concept, methodology, and use for participatory needs assessment. Heal Educ Behav. 1997;24:369–87.
21. Li VC, Wang S, Wu K, Zhang W, Buchthal O, Wong GC, et al. Capacity building to improve women’s health in rural China. Women’s Reprod. Heal. Dev. Progr. 2001;52:279–92.
22. Budig K, Diez J, Conde P, Sastre M, Hernán M, Franco M. Photovoice and empowerment: evaluating the transformative potential of a participatory action research project. BMC public health. 2018 Dec;18(1):1-9.
23. Israel BA, Coombe CM, Cheezum RR, Schulz AJ, McGranaghan RJ, Lichtenstein R, et al. Community-based participatory research: a capacity-building approach for policy advocacy aimed at eliminating health disparities. Am. J. Public Health. 2010;100:2094–102.
24. Saidu S (2021) (PhD student – Photovoice Personal communication 9 Nov 2021)
25. Hergenrather KC, Rhodes SD, Clark G. Windows to work: exploring employment-seeking behaviors of persons with HIV/AIDS through photovoice. AIDS Educ. Prev. 2006;18:243–58.
26. BOOTH T, BOOTH W. In the Frame: photovoice and mothers with learning difficulties. Disabil. Soc. 2003;18:431–42.
27. Castleden H, Garvin T, First Nation H ay aht. Modifying Photovoice for community-based participatory Indigenous research. Soc. Sci. Med. 2008;66:1393–405.
28. Ardrey J, Desmond N, Tolhurst R, Mortimer K. The Cooking and Pneumonia Study (CAPS) in Malawi: A nested pilot of photovoice participatory research methodology. PloS one. 2016 Jun 2;11(6):e0156500.

# Appendix 1: 2YLs prospective sites

| **Town/ village** | **Chiefdom** | **District** | **Name of PHU** | **Number of SVD births in 2020** | **2020-Total number of biths (SVD, assisted, CS), all ages** | **2020-Total number of biths (SVD, assisted, CS), <19** | **Rate** | **Nearest hospital for CS** | **Distance/**  **time taken to travel to hospital** | **Site number for randomisation** |
| --- | --- | --- | --- | --- | --- | --- | --- | --- | --- | --- |
| Moriba Town | Lower Banta Chiefdom | Moyamba | Moriba Town (Lower Banta) CHP | 147 | 135 | 55  (4-6 p/month) | 33% | Moyamba Government Hospital | 71km,  1hr 53 min | 1b |
| Mattru Town CHC | Jong chiefdom | Bonthe | Mattru Town CHC | 67 | 60 | 28  (2-3 p/month) | 64% | Bo Government Hospital | 81km,  2hrs, 3 min | 2a |
| Madina | Bum chiefdom | Bonthe | Madina (Bum) CHC | 121 | 100 | 39  (3-6 p/month) | 46% | Bo Government Hospital | 125km,  2hrs, 47 min | 2b |
| Senehun | Kamajei chiefdom | Moyamba | Senehun (Kamajei) CHC | 95 | 77 | 30  (2-6 p/month) | 60% | Bo Government Hospital | 38km,  31 min | 3b |
| Taiama | Kori Chiefdom | Moyamba | Taiama (Kori) CHC | 102 | 99 | 38  (2-5 p/month) | 47% | Bo Government Hospital | 51km,  43 minutes | 4a |
| Moyamba Junction | Fakunya chiefdom | Moyamba | Moyamba Junction CHC | 115 | 102 | 45  (3-6 p/month) | 40% | Moyamba Government Hospital | 34km,  31 minutes | 4b |
| Lengekoro | Diang chiefdom | Koinadugu | Lengekoro CHP | 112 | 97 | 41  (3-5 p/month) | 44% | Kabala Government Hospital | 22 km,  22 minutes | 5a |
| Heremakono | Wara wara yagala chiefdom | Koinadugu | Heremakono MCHP | 118 | 123 | 56  (4-6 p/month) | 32% | Kabala Government Hospital | 12 km,  14 minutes | 5b |
| Yataya | Wara wara yagala chiefdom | Koinadugu | Yataya CHP | 90 | 78 | 28  (2-4 p/month) | 64% | Kabala Government Hospital | 6km, 14 minutes | 6b |
| Tombo | York rural zone | Western Rural | Tombo town (York rural) CHC | 119 | 100 | 49  (variable, some months >10 others <4 or none?) | 37% | PCMH | 42 km,  1hr, 3 minutes | 7a |
| Kaningo | West 3 Zone | Western Urban | PAYCY's Clinic | 147 | 157 | 65  (6-10 per month) | 28% | PCMH | 5km,  20 minutes | 8a |
| Calaba Town | East 3 | Western Urban | Calaba Town CHC | 90 | 79 | 30  (3-5 per month) | 60% | PCMH | 3km,  12 minutes | 8b |
|  |  |  |  |  |  |  | 45.5% |  |  |  |
